# Funding global emergency medicine research—from seed grants to NIH support

**DOI:** 10.1186/s12245-016-0121-8

**Published:** 2016-10-18

**Authors:** Bhakti Hansoti, Adam Levine, Latha Ganti, Rockefeller Oteng, Taylor DesRosiers, Payal Modi, Jeremy Brown

**Affiliations:** 1 Department of Emergency Medicine, Johns Hopkins Hospital, Baltimore, MD USA; 2 Department of Emergency Medicine, Brown University, Providence, RI USA; 3 Department of Emergency Medicine, University of Central Florida, Orlando, FL USA; 4 Department of Emergency Medicine, University of Michigan, Ann Arbor, MI 48105 USA; 5 Department of Emergency Medicine, Johns Hopkins Hospital, Baltimore, MD 21287 USA; 6 Emergency Medicine, University of Massachusetts, Worcester, MA USA; 7 The National Institutes of Health, Office of Emergency Care Research, Bethesda, MD USA

**Keywords:** Public Health, Global Emergency Medicine, International Emergency Medicine, Research, Funding, Grants, Emergency care

## Abstract

**Background:**

Funding for global health has grown significantly over the past two decades. Numerous funding opportunities for international development and research work exist; however, they can be difficult to navigate. The 2013 Academic Emergency Medicine consensus conference on global health and emergency care identified the need to strengthen global emergency care research funding, solidify existing funding streams, and expand funding sources.

**Results:**

This piece focuses on the various federal funding opportunities available to support emergency physicians conducting international research from seed funding to large institutional grants. In particular, we focus on the application and review processes for the Fulbright and Fogarty programs, National Institutes of Health (NIH) Career development awards, and the Medical Education Partnership Initiative (MEPI), including tips and pathways through each application process.

**Conclusions:**

Lastly, the paper provides an index that may be used as a guide in determining whether the amount of funding provided by a grant is worth the effort in applying.

## Background

Funding for global health has grown significantly over the past two decades. In 1990, an estimated US$5.6 billion was spent on development assistance for health while in 2011, estimates demonstrated a spending increase upwards of US$27.7 billion [1]. While most funding has traditionally been earmarked for program development and implementation, there has been a steady growth in global health research funding as well. Unfortunately, much of this research funding has been directed towards specific disease processes and little has been focused on emergency care research.

The 2013 Academic Emergency Medicine consensus conference on global health and emergency care identified the need to strengthen global emergency care research funding, solidify existing funding streams, and expand funding sources [2]. These goals focused on quantifying the funding opportunities for global health and emergency care research, improving understanding of current research priorities and identifying barriers to research funding.

The demands on emergency medicine services nationally and internationally continue to grow [3, 4]. The mission of many NIH institutes includes improving the health of persons with conditions that commonly present to the emergency department (ED), and the NIH is committed to continued support of emergency care research [5]. On July 31st of 2012, the NIH announced the formation of the Office of Emergency Care Research (OECR) with the intention of creating a focal point for emergency care research and training across the NIH [6]. The office assists applicants to obtain funding and promotes emergency care research across the NIH. The OECR maintains a list of funding opportunities for emergency care research on its website [7]. This list of research opportunities attempts to consolidate emergency care oriented, NIH-supported, funding prospects, and may provide a starting point for researchers looking for funding opportunities [8].

The National Institutes of Health (NIH) is a biomedical research facility and agency of the United States Department of Human Health and Services. The NIH conducts its own research through its Intramural Research Program (IRP) and provides a great deal of research funding through its Extramural Research Program. There are 27 separate institutes and centers housed under the NIH, and all support some international research. However, the FIC is the only institute within the 27 with global health as a primary mission [9].

Numerous funding opportunities for international development and research work exist; however, they can be difficult to navigate. This piece focuses on the various federal funding opportunities available to support emergency physicians conducting international research. We attempt to create a guide to independent funding for global emergency medicine researchers, starting with seed funding all the way to large institutional grants. In particular, we focus on the application and review processes for the Fulbright and Fogarty programs, National Institutes of Health (NIH) Career development awards, and the Medical Education Partnership Initiative (MEPI) [10].

## Before federal funding: seed grants

Prior funding is the biggest advantage when attempting to secure future funding. Hence, applying for seed grant funding for a smaller project or pilot project is advisable prior to applying for federal funding. Non-governmental foundations tend to be an excellent and often overlooked source of funding for junior researchers, especially the Doris Duke Foundation in the USA and the Wellcome Trust in the UK [11].

Seed grant funding is appropriate for researchers of all levels to apply for, including medical students, residents, junior researchers, as well as junior faculty. Each grant varies greatly in type, amount, and expected product, allowing the applicant to strategically apply for a range of funding.

The Bill and Melinda Gates Foundation remains the largest private funder of global health research in the world [12]. While most of its research awards are large-scale grants to established researchers and organizations, the Gates Foundation Grand Challenges Exploration grants are smaller, $100,000 awards for innovative pilot programs on a variety of global health topics. These awards have several funding cycles each year and generally require a short, two-page application to get started.

The Humanitarian Innovation Fund and Research for Health in Humanitarian Crises (R2HC) offer both large and small grants for research directly related to disaster and humanitarian response. The Thrasher Foundation awards a number of grants each year for global health research related to children’s health, especially their $25,000 Early Career Award specifically targeting junior researchers.

Emergency medicine organizations have also recognized the need for seed funding in order to ensure the long-term success of their members that choose a career in global emergency medicine research. Thus, the Emergency Medicine Foundation (EMF) recently established a $10,000 grant specific to International Emergency Medicine research projects, and the Society of Academic Emergency Medicine Foundation (SAEMF) followed suite establishing global emergency medicine research grants starting 2015 in addition to their well-established research training grant for junior faculty [13, 14]. Lastly, the Emergency Medicine Residents Association has several smaller research grants available to emergency medicine residents, which can be used for international projects [15].

In addition to foundation grants, industry-sponsored awards are another potential source of seed grant funding. Industry partners, including drug and medical device makers, may sponsor a research project, donate equipment, or provide valuable contacts to conduct research. Seed grants for global health research projects may also be available locally, either through a university, hospital, or academic department. While these tend to be smaller grants, they are often easier to procure due to a smaller pool of applicants and can be an excellent first step on the long journey to becoming an independently funded, global emergency medicine researcher.

Tip: Funding attracts more funding, explore far and wide for seed grants

## Fogarty and Fulbright fellowships

Fellowships are the next level of grant funding and provide the opportunity for a more longitudinal combined training and research experience. The goal of many of these fellowship awards is to help establish (a) a mentorship team and (b) provide preliminary data for a career development award. The Fogarty International Clinical Research Scholars (FICRS) program, established in 2004, was focused on tropical medicine training and was successful in training researchers with expertise in communicable and non-communicable disease worldwide. In 2012, the program was restructured as the Global Health Training for Fellows and Scholars (GHTFS) and is now administered by five university support centers. The new program shifts the emphasis from students to post-doctoral trainees with the goal to develop advanced researchers capable of obtaining independent research support [16]. The program provides year-long research training experiences for early stages investigators with terminal degrees from the USA as well as low- or middle-income host country (LMIC) trainees at international sites, typically research centers and/or universities participating in the FIC training programs [17].

The Fulbright program is the second side of the Fulbright-Fogarty relationship and was created in 1946 by Senator J. William Fulbright of Arkansas to establish an international exchange program with the goal to “increase mutual understanding between the people of the United States and the people of other countries” [18]. A Fulbright scholarship is designed for people at varying stages in their careers with terms ranging from 2–11 months. One does not need a terminal degree to apply for a Fulbright scholarship, unlike the Global Health Training for Fellows and Scholars program. The Fulbright faculty program sends approximately 800 US professors, scholars, and professionals to 125 countries to teach, conduct research, share their expertise, and promote goodwill between countries [19].

In general, both Fogarty and Fulbright fellowships provide financial support for travel and research, as well as a living stipend. Salary support is negotiable and tends to be at a post-graduate year (PGY) 4 level. While Fogarty fellowships are only for research projects, Fulbright’s can be for teaching or a combination of research and teaching. Both are generally targeted at junior faculty or residents wishing to engage in an immersion global health experience through a short application process (Table 1).

**Table 1:** Fogarty and Fulbright application outline

1. Basic personal data 2. Statement of grant purpose Research/study: 2 pages Budget/timeline: 1 page Personal Statement: 1 page 3. Report/references Foreign language Three references (includes one from the department chair ensuring clinical time off) 4. Transcripts (Fulbright only)	

Tip: The biggest advantage to both of these programs is that they give you access to a world of mentors and collaborators in this field, and are also a good stepping stone to gather pilot data and secure future grant funding.

## Career development awards

The NIH supports career advancement through NIH Career Development Awards, also known as K awards by the first letter of their grant number (i.e., K08 or K23). These awards traditionally serve as the first rung on the NIH ladder, targeted towards junior faculty in the first 5 to 7 years after completing their final training program. Similarly to other Fogarty programs, the goal of awarding K awards is to help junior faculty develop both their research and academic careers so they prepare to apply for a standard NIH grant, such as an R01 award [20].

The K awards are 3 to 5 years in length, though most physicians choose 5-year awards. They provide $75,000 in salary support for the principal investigator (plus extra support to cover fringe benefits) and $25,000–$40,000 per year for research, training, and travel costs depending on the specific NIH agency. The K award comes with the expectation that the principal investigator will spend 75 % of their time dedicated to their research with the remaining 25 % of the time spent on clinical, teaching, and administrative responsibilities. Since $75,000 per year generally does not cover 75 % of a junior faculty’s salary, K awards generally require the principal investigator’s department or institution to provide some additional support to make up the difference. Thus, the likelihood of success and the support required to secure a K award is heavily dependent on the applicant’s academic institution [21].

A recent study found that between 2000 and 2011, 63 K awards were granted to emergency physicians. While only a small percentage of total awards during those years, the overall success of those applying from an emergency medicine background has increased from 2–4 awards per year from 2000 to 2005 to 5–11 awards per year from 2006 to 2011 [22].

NIH agencies fund a number of different types of K awards each year, including the K08 award targeted towards basic science research and the K23 award targeted towards clinical and operational research. The Fogarty International Center administers the K01 award, also called the International Research Scientist Development Award (IRSDA), targeted towards international researchers and requiring the applicant to spend at least 50 % of their time at the international site. It also requires that the applicant have dedicated mentorship from both their home institution and their international project site. A special panel of global health experts who are more likely to understand the logistical constraints and nuances of conducting global health research review the FIC K01 awards [23].

While all NIH agencies can fund international projects, the Fogarty International Center (FIC), the National Institute of Allergy and Infectious Disease (NIAID), and the National Institute of Child Health and Development (NICHD) tend to fund more international projects. The application for a K award can be long and tedious with multiple components, and often takes at least 6–9 months of preparation (Table 2) [22].

**Table 2:** Components of a K award

• Project summary/public health statement (1 page) • Specific aims (1 page) • Combined sections (12 pages total) • Candidate BACKGROUND (1–2) • Career goals and objectives (1–2) • Development activities during award (2–3) • Responsible conduct of research (0.5) • Research strategy (6-8) • Institutional environment (1 page) • Protection of human subjects (4–5 pages) • Mentor letters (1–3 letters) • Reference letters (3–5 letters) • Institutional commitment letter • Biosketches for candidate and mentors (4 pages each in NIH format) • Budget and budget justification • Facilities (2–3 pages) • Inclusion of women and minorities (1 page) • Inclusion of children (1 page)	

There are several important keys to success when preparing an NIH K award application. Since the K award is different than most other NIH grants in that they are considered “mentored” awards, the first step is to identify strong mentors. The principal investigator is expected to have one or more mentors who have topical and/or geographic expertise relevant to the research project, who will guide them through the process of conducting the research project. In addition, the primary mentor should be the recipient of prior NIH research grants and ideally, the candidate’s project will fit in as a sub-study or a follow-up study to their mentor’s research. The mentor does not have to be an emergency physician—three quarters of prior emergency medicine K awardees had mentors outside of emergency medicine. The candidate should have a few prior publications, ideally in their research area, in addition to the ongoing grant funding, usually from a department or foundation grant.

Before applying for a K award, the applicant should read the Funding Opportunity Announcement carefully to make sure they meet all the necessary requirements. It is especially important to review the section titled “Application Review Criteria” which includes the actual questions given to reviewers when evaluating the applications. It is essential that the applicant clearly and concisely answer each of these questions in order to increase the likelihood of being assigned a good score. In addition, each NIH agency will have a specific program officer assigned to work with their K award recipients and applicants. Talking with the program officer in advance can also be helpful to ensure that the project fits in with the mission and funding interests of the agency.

Tip: K awards can help junior faculty develop their research and academic careers, increasing the likelihood of success when applying for standard NIH grants, such as an R01 award. Of the 60 % emergency medicine awardees who completed their K award by 2011, 42 % obtained subsequent federal funding with 16 % obtaining R01 funding as well. The median time from the end of K award to receipt of an R01 award was 4.5 years [22].

## Independent funding

The R Series of NIH grants, while not specific to the Fogarty International Center, are available for research-related endeavors. For example, the R01 is the NIH’s most commonly used grant program, and is used to support discrete research projects in all fields. Another example is the R25 that is awarded for education projects to support the development or implementation of a program in the areas of education, information, training, technical assistance, coordination, or evaluation. Both of these awards, as well as the exhaustive list of all R grants, can be used in a variety of ways by all fields at almost all levels to support research or resources for research. R awards are predominantly given to those with robust prior research and experience. Thus applicants must demonstrate robust prior training in clinical research, as there is no mentorship component to the R award applications.

Tip: As mentioned above, many of the NIH support overseas research, but do so in their mission areas. Potential applicants should be sure that their project is within the mission areas of the parent institute. A strong application that does not fall squarely within the mission of a specific institute will have difficulty being funded.

## Fogarty International Center and Research Training Grants

While the John E. Fogarty International Center (FIC) does not work exclusively on emergency medicine, their yearly budget of $69 million funds their mission to support and facilitate global health research conducted by USA and international investigators. Furthermore, the stated objective of the organization is to help communities in low- and middle-income countries (LMICs) sustain the training of future generations of researchers and scientists committed to exploring and solving the health challenges of their home countries, with a focus on non-communicable and chronic diseases. The current Fogarty director Dr. Roger Glass stated that, “Only by building partnerships with researchers overseas will the United States be able to maintain its competitive edge and accelerate the expansion of knowledge for understanding and the cures desired by all” (Table 3).

**Table 3:** Fogarty International Center Strategic Plan 2014 [23]

The FIC published its most recent strategic plan in March 2014, with the five following goals:
• Build research capacity through Individuals, Institutions, and Networks to meet future and evolving global health challenges. • Stimulate innovation in the development and implementation of technologies and other locally relevant solutions to address global health problems. • Support research and research training in implementation science. • Advance research on prevention and control of the dual burden of communicable and non-communicable diseases and disabilities. • Build and strengthen partnerships to advance global health research and research capacity.

In 2013, the FIC issued over $16,000,000 of new grant support to international projects. Various grants are available based on the spirit of the project; the following represent a non-exhaustive list of examples. The most frequently awarded grant is the D43, an International Research Training Grant unique to Fogarty International Center. The purpose of the D43 is to support research-training programs for both US and foreign professionals and students to strengthen global health research and collaboration. This grant would be appropriate for someone who is attempting to establish long-term international research collaboration with capacity building. To help prepare for an application for a D43, the D71 International Research Training Planning Grant is available as the second most commonly award FIC grant. This differs from the D71 by emphasizing the planning rather than execution of a research project. This grant would be appropriate for planning a larger international research project in low- or middle-income countries. Examples of recently funded D43 projects are a project in Ghana on mental health, a training program in cancer biomarkers in Thailand, a training program to prevent HIV infection in women in South Africa, and a program to build junior research faculty in Ethiopia.

The overall success rate when considering all applications for all grants and awards is 33 %. Between 2009 and 2013, Fogarty received 17 new submissions from emergency medicine department affiliated primary investigator’s (PI), of which only four were successful in receiving funding.

Tip: Before considering a submission, it is important to understand and review the Fogarty Strategic plan and reflect how your grant proposal supports or reflects the goals of the FIC.

## Institutional funding: Medical Education Partnership Initiative (MEPI)

The MEPI is a unique funding stream available to those with an interest in medical education and research expansion and warrants special mention, as it directed towards development in Sub-Saharan Africa. The MEPI initiative was created with the purpose of funding foreign institutions and their partners in sub-Saharan Africa to develop, expand, and enhance models of medical education. Targeting countries that receive PEPFAR (acronym for the President’s Emergency Plan for AIDS Relief) support, MEPI’s goal is to increase the number of new healthcare workers, strengthen in-country medical education systems, and build clinical and research capacity [24]. This unique approach of having the African institution serve as the principal investigator is meant for recipients to aid in the development of an in-country program that fosters retention of personnel and project outcomes. The Office of the US Global AIDS Coordinator (OGAC) largely funds the MEPI program. FIC/NIH and the HIV/AIDS Bureau of the Health Resources and Services Administration (GHAP/HAB/HRSA) jointly coordinate the administration of the MEPI program.

MEPI awards are institutional awards, with collaboration across specialties and are best suited for those who wish to work in the sphere of education or research infrastructure expansion. The awardees are expected to work within country to establish long-lasting frameworks that will remain after the closure of their individual project. The call for MEPI partners emphasizes the organization mission to support African institutions, strengthening human capacity by fighting brain drain and expanding medical education (Table 4). Given the lack of human resources for health in Africa, a workforce trained to address and understand (research) this burden would then fit the call [25].

**Table 4:** MEPI overview [25]

There are 3 levels of awards that can be proposed
• Awards focused on PEPFAR priority areas (programmatic awards: $2 million/year) • Linked programmatic awards that support non-communicable diseases and priority health areas related to and/or beyond HIV/AIDS (linked awards: $500,000/year), and a • Coordinating center ($2 million/year)

Tip: The MEPI program has several specific directions that should be kept in mind when considering this as a potential funding pathway. Proposals must seek to expand and/or enhance innovative medical education models that have the potential to improve the quality of clinical education and clinical care in countries in Sub-Saharan Africa and develop strategies that enable graduating medical students to remain in their home country to practice, serve as faculty, and/or conduct research related to the implementation of PEPFAR and other public health priorities. Proposal must seek to enhance the recruitment and retention of qualified academic faculty through partnerships and research opportunities.

## The numbers game

Applying for grants is partly a numbers game; the more grants you submit, the more likely you are to be awarded funding. Even so, it is still important to be somewhat selective in deciding which grants to submit. An applicant should start by reading the philosophy of the granting organization and reviewing the lists of prior awardees to see if they align with the applicant’s own research interests.

It is also important to review the grant instructions carefully to make sure the application includes all the necessary supporting information, falls within the defined word or page limit, and is formatted correctly. Talking with prior grant awardees can also be helpful to provide information on what the reviewers are looking for in a project and other relevant details.

Table 5 demonstrates an index, developed by Dr. Adam Levine that may be used as a guide in determining whether the amount of funding provided by a grant is worth the effort in applying.

**Table 5:** Levine index

• $$ \mathrm{Levine}\ \mathrm{Index} = \frac{\left(\mathrm{Percent}\ \mathrm{o}\mathrm{f}\ \mathrm{Grant}\mathrm{s}\ \mathrm{Awarded}\right)\times \left(\mathrm{Total}\ \mathrm{Grant}\ \mathrm{Amount}\ \mathrm{in}\ \mathrm{Dollars}\right)}{\left(\mathrm{Total}\ \mathrm{Hours}\ \mathrm{t}\mathrm{o}\ \mathrm{Complete}\ \mathrm{Application}\right)} $$ • $$ \mathrm{Modified}\ \mathrm{L}\mathrm{evine}\ \mathrm{I}\mathrm{ndex}\ \left(\mathrm{M}\mathrm{L}\mathrm{I}\right) = \frac{\left(\mathrm{Percent}\ \mathrm{of}\ \mathrm{Grant}\mathrm{s}\ \mathrm{Awarded}\right)\times \left(\mathrm{Total}\ \mathrm{Grant}\ \mathrm{Amount}\ \mathrm{in}\ \mathrm{Dollars}\right)}{\left(\mathrm{Total}\ \mathrm{Number}\ \mathrm{of}\ \mathrm{Application}\ \mathrm{Pages} \times 10\right)} $$ • A $100,000 grant with 8 applicants per year for two awards (or a percent awarded of 0.25) that has a 20 page application would have an MLI = 125 • Alternatively, a $10,000 grant with 10 applicants for one award (or a percent awarded of .10) and a 15 page application would have an MLI = 7 • A “good” MLI will depend on how much a researcher’s time is worth—for a junior researcher, an MLI of >50 might be fine, while a very senior researcher may only find it worthwhile to apply for grants with an MLI >500. Regardless, the MLI should only be one of several criteria used to evaluate whether a specific grant is the right fit for a given applicant.	

## Conclusions

**Fig. 1:**
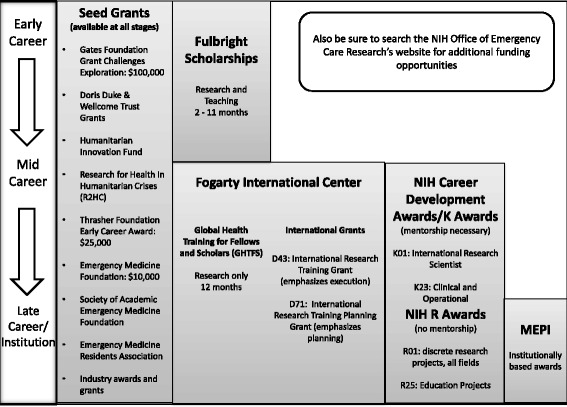
Infographic on timeline of grant applications

There are numerous funding pathways for global health. The key to success for junior faculty is to identify smaller awards such as seed grants or fellowships to get pilot data, experience and establish mentorship/collaborative relationships. It is necessary with all funding awards to have established strong collaborative relationships with colleagues in the field (especially for the MEPI program). The breadth of emergency medicine allows for researchers to seek funding from many different sources. This is both an advantage and disadvantage. Most importantly funding begets more funding, thus global emergency medicine researchers must work together to produce projects, protocols, and peer networks to develop opportunities in this important field (Fig. 1).

## Useful links

NIH Common Fund/Office of the Director (OD/OSC/CF/NIH)

Office of AIDS Research, Office of the Director (OAR/NIH)

Office of Research on Women’s Health (ORWH/NIH)

National Heart, Lung, and Blood Institute (NHLBI/NIH)

National Human Genome Research Institute (NHGRI/NIH)

National Institute of Mental Health (NIMH/NIH)

National Institute of Neurological Disorders and Stroke (NINDS/NIH)

National Institute of Nursing Research (NINR/NIH)

Fulbright US

Fulbright State

## References

[1] Lancet (2009). Who runs global health?. Lancet.

[2] Hansoti B, Weiner S, Martin I (2013). Society for Academic Emergency Medicine’s Global Emergency Medicine Academy: Global Health Elective Code of Conduct. Acad Emerg Med.

[3] Glickman S, Kit Delgado M, Hirshon J (2010). Defining and measuring successful emergency care networks: a research agenda. Acad Emerg Med.

[4] Razzak JA, Kellermann AL (2002). Emergency medical care in developing countries: is it worthwhile?. Bull World Health Organ.

[5] Cairns C, Maier R, Adeoye O (2010). NIH roundtable on emergency trauma research. Ann Emerg Med.

[6] Mitka M (2012). NIH signals intent to boost funding of emergency care research and training. JAMA.

[7] Nigms.nih.gov. Office of Emergency Care Research - National Institute of General Medical Sciences. http://www.nigms.nih.gov/about/overview/oecr/Pages/default.aspx. Accessed 30 Sept 2015.

[8] Office of emergency care research, funding opportunities. http://www.nigms.nih.gov/about/overview/OECR/Pages/funding.aspx. Accessed 17 Jun 2015.

[9] NIH organizational chart http://oma.od.nih.gov/public/MS/organization/Organization%20Charts/OD-NIH.pdf. Accessed 17 Jun 2015.

[10] Doney M, Smith J, Kapur B (2005). Funding emergency medicine development in low- and middle-income countries. Emerg Med Clin N Am.

[11] Kaiser J (2009). Wellcome Trust to shift from projects to people. Science.

[12] Foundationcenter.org. Foundation Center - About FC Stats. http://foundationcenter.org/. Accessed 29 Sept 2015.

[13] Emfoundation.org. Emergency Medicine Foundation.http://www.emfoundation.org/applyforagrant/. Accessed 30 Sept 2015.

[14] Saem.org. Grants. http://www.saem.org/saem-foundation/grants. Accessed 30 Sept 2015.

[15] Emra.org. EMRA Research Grant. http://www.emra.org/content.aspx?id=167. Accessed 30 Sept 2015.

[16] Benziger CP, Gilman RH (2014). The impact of the Fogarty International Clinical Scholars and Fellows program extends beyond borders. Am J Trop Med Hyg.

[17] Carothers C, Heimburger D, Schlachter S (2013). Training programs within global networks: lessons learned in the Fogarty International Clinical Research Scholars and Fellows Program. Am J Trop Med Hyg.

[18] Cottrell RR (2012). Fulbright programs: an opportunity for career development. Health Promot Pract.

[19] Breman J, Bridbord K, Kupfer L, Glass R (2011). Global Health: The Fogarty International Center, National Institutes of Health: vision and mission, programs, and accomplishments. Infect Dis Clin N Am.

[20] Nichd.nih.gov. Career Development (K) Awards. https://www.nichd.nih.gov/training/extramural/Pages/career.aspx. Accessed 30 Sept 2015.

[21] Grants.nih.gov. Mentored clinical scientist research career development award (Parent K08) (PA-14-046). http://grants.nih.gov/grants/guide/contacts/parent_K08.html. Accessed 30 Sept 2015.

[22] Nishijima D, Yadav K, May L, Kraynov L, Courtney DM (2013). Description and productivity of emergency medicine researchers receiving K23 or K08 mentored research career development awards. Acad Emerg Med.

[23] Voelker R (2008). Fogarty at 40: NIH center updates its strategies for supporting global health. JAMA.

[24] Chen L, Evans T, Anand S (2004). Human resources for health: overcoming the crisis. Lancet.

[25] Medical Education Partnership Initiative (MEPI). http://www.fic.nih.gov/programs/pages/medical-education-africa.aspx. Accessed 14 Aug 2015.

